# OXPHOS mediators in acute myeloid leukemia patients: Prognostic biomarkers and therapeutic targets for personalized medicine

**DOI:** 10.1186/s12957-024-03581-5

**Published:** 2024-11-12

**Authors:** Amal Kamal Abdel-Aziz

**Affiliations:** Department of Pharmacology and Toxicology, Faculty of Pharmacy, Ain Shams University, Abbassia, Cairo, 11566 Egypt

**Keywords:** Acute myeloid leukemia, Mitochondrial biomarker, Oxidative phosphorylation, Prognosis

## Abstract

**Background:**

Despite significant advances in comprehending its tumorigenic role, the prognostic and therapeutic potential of targeting oxidative phosphorylation (OXPHOS) in acute myeloid leukemia (AML) remain obscure.

**Methods:**

The prognostic value of ~ 200 mitochondrial/OXPHOS genes as candidate biomarkers was examined in AML patients over ~ 10 years follow-up using Kaplan–Meier and Cox regression analyses. Furthermore, the transcript levels of the assessed markers were inspected in healthy bone marrow tissues and the dependencies of AML cells on the assessed genes were examined.

**Results:**

Elevated levels of NADH:ubiquinone oxidoreductase subunit A6 (NDUFA6), succinate dehydrogenase complex flavoprotein subunit A (SDHA), solute carrier family 25 member 12 (SLC25A12), electron transfer flavoprotein subunit beta (ETFB), carnitine palmitoyltransferase 1A (CPT1A) and glutathione peroxidase 4 (GPX4) were associated with poor overall survival of AML patients. SLC25A12, ETFB and CPT1A were overexpressed in AML compared to healthy tissues. Cytochrome B5 type A (CYB5A)^high^, SLC25A12^high^ and GPX4^high^ AML patients displayed higher levels of circulating and engrafted blasts compared to low-expressing cohorts. NPM1 and SRSF2 mutations were frequent in SDHA^low^ and CPT1A^low^ AML patients respectively. FLT3-ITD, NPM1 and IDH1 mutations were prevalent in CPT1A^high^ AML patients. FLT3-ITD AMLs were more dependent on OXPHOS.

**Conclusions:**

This study identifies NDUFA6 and SDHA as novel companion prognostic biomarkers which might present a rational strategy for personalized therapy of AML patients.

**Graphical Abstract:**

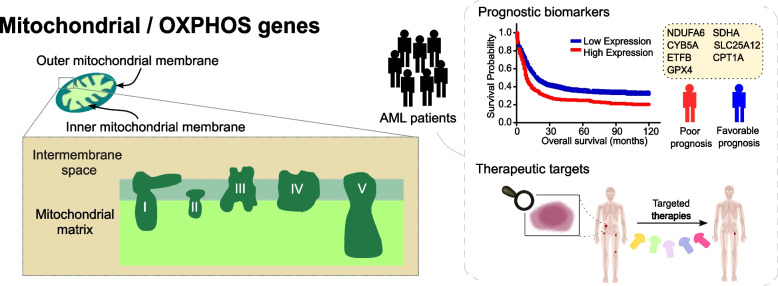

**Supplementary Information:**

The online version contains supplementary material available at 10.1186/s12957-024-03581-5.

## Background

Acute myeloid leukemia (AML) is an aggressive type of blood cancer which is characterized by impaired differentiation and unlimited proliferation of AML blasts [1, 2]. Despite remarkable advances in treating other types of cancer, conventional chemotherapy remained the main standard of care therapy for AML patients [2–4]. With the advent of cutting-edge genomics technologies, genetic mutations were identified as fundamental players contributing to AML pathogenesis [1, 2, 5, 6]. Of note, multiple genetic lesions might co-exist in the same AML patient. Nucleophosmin 1 (NPM1) mutation is one of the common AML mutations which is associated with abnormal cytoplasmic localization of NPM1 [7]. Isocitrate dehydrogenase 1 (IDH1) and isocitrate dehydrogenase 2 (IDH2) mutations account for ~ 15–25% of AML mutations. Mutated IDH1 and IDH2 trigger the production of 2-hydroxyglutarate oncometabolite which is associated with defective hematopoietic differentiation [8]. Mutations in *CCAAT enhancer binding protein A gene* (*CEBPA*), a crucial transcription factor for the differentiation of granulocytes, are also among the frequent AML mutations. FMS-like tyrosine kinase 3 (FLT3) internal tandem duplication (FLT3-ITD) mutations and FLT3 tyrosine kinase domain (FLT3-TKD) mutations promote constitutive activation of FLT3 signaling which ultimately favour the proliferation of AML cells [5]. Over the last decades, several targeted agents as isocitrate dehydrogenase (IDH) inhibitors, fms-like tyrosine kinase 3 (FLT3) inhibitors, B-cell lymphoma 2 (BCL2) inhibitors, hypomethylating agents and menin inhibitors were developed and some were approved for AML therapy [5, 9–17].

Several studies underscored the role of oxidative phosphorylation (OXPHOS) in mediating the pathogenesis as well as resistance of diverse types of cancer including AML [18–21]. To meet their heightened demands of energy and biosynthetic precursors, cancer cells extensively metabolize substrates as glucose, glutamine and fatty acids to ultimately generate electrons which transfer across mitochondrial complexes [18]. Co-culturing AML cells with bone marrow derived stromal cells triggered OXPHOS and mitochondrial ATP synthesis in AML cells which exhibited chemoresistance [20]. Indeed, OXPHOS signature was enriched in cytarabine-resistant persisting AML cells [22]. Targeting mitochondrial protein synthesis or electron transfer enhanced the anti-leukemic activity of cytarabine [22]. Pharmacological inhibition of mitochondrial OXPHOS exhibited potent anticancer activities [23]. Of note, AML cells are vulnerable to inhibitors of the respiratory chain complexes as well as oxidative stressors [24]. Via inhibiting mitochondrial complex I, mubritinib elicited strong anti-AML effects in vitro and in vivo [25]. Tackling AML heterogeneity (including leukemic stem cells (LSCs) + more differentiated/mature AML blasts) is fundamental for eradicating AML [26–29]. Unlike hematopoietic stem cells (HSCs) which rely on glycolysis, LSCs are OXPHOS-dependent [30]. The imipridone ONC213 demonstrated potent anti-AML activity, particularly in LSCs, via inhibiting α-ketoglutarate dehydrogenase which impeded OXPHOS [31]. Durable remissions of AML patients who were treated with venetoclax (a selective BCL2 inhibitor) and azacitidine (a hypomethylating agent) were linked to inhibition of electron transport chain (ETC) complex II and hence OXPHOS suppression which preferentially targeted LSCs [32].

According to the NCI’s Cancer Stat Facts, the 5-year survival rate of AML patients is approximately 32%. Development of reliable and sensitive prognostic biomarkers is critically warranted for stratifying AML patients into low-risk and high-risk cohorts and guiding clinicians towards rational “tolerable and efficacious” therapeutic regimens. Diverse methods are proposed to assess mitochondrial oxygen consumption [33, 34]. Eight mitochondrial biomarkers predicted the progression and poor overall survival (OS) of gastric cancer patients [35]. More studies are needed to assess the prognostic potential of OXPHOS genes in AML patients. Indeed, this study aimed at systematically investigating the prognostic value of OXPHOS genes as candidate biomarkers to predict the clinical outcome of AML patients.

## Materials and methods

### Kaplan–Meier (K-M) survival analyses

To perform Kaplan–Meier (K-M) analysis on OXPHOS transcripts, the list of human genes encoding proteins involved in oxidative phosphorylation (HALLMARK_OXIDATIVE_PHOSPHORYLATION) was downloaded from GSEA MSigDB (https://www.gsea-msigdb.org/gsea/msigdb/human/geneset/HALLMARK_OXIDATIVE_PHOSPHORYLATION.html.). Hazard-ratios (HR) for the overall survival (OS) and event-free survival (EFS) were calculated, with 120 months (~ 10 years) follow-up threshold at the best auto-selected cut-off on AML datasets (GSE1159, GSE6891, GSE8970, GSE12417 and GSE37642) and *P*-values were calculated using the log-rank test. K-M curves were also generated online by the K-M-plotter, using univariate analysis. False discovery rate (FDR) was computed using Benjamini–Hochberg method as described (https://kmplot.com/analysis/index.php?p=service&cancer=aml). Genes linked to poor prognosis (i.e. HR > 1) were selected for further evaluation given their therapeutic potential. For the filtered genes, multivariate Cox regression was exploited to validate the potential effect of clinical variables and gene expression on the survival of AML patients. K-M analyses were also restricted to AML patients with IDH1, IDH2, NPM1, FLT3-ITD, FLT3-TKD and CEBPA mutations. The most updated version of the database was exploited for this analysis [36].

### Gene Expression Profiling Interactive Analysis (GEPIA)

Gene Expression Profiling Interactive Analysis (GEPIA) carries out OS analysis based on the gene expression in biospecimens obtained from AML patients [37]. In this study, we investigated the potential candidacy of mitochondrial / OXPHOS mediators as prognostic biomarkers for AML patients. To this end, HRs were calculated based on Cox proportional-hazards model at the median cut-off, and *P*-values were calculated using the logrank test [37].

### cBioportal – OS analysis

To validate the results obtained from Km plotter and GEPIA database and inspect the effects of the expression of the indicated genes on the OS of AML patients, RNA-seq, clinical and survival data of BeatAML.2 dataset (OHSU, Cancer Cell, 2022) were downloaded from cBioPortal [38]. HR for the OS were calculated, with 120 months (~ 10 years) follow-up threshold at the best auto-selected cut-off and *P*-values were calculated using the log-rank test. The receiver operating characteristic (ROC) curves and all statistical computations were executed to evaluate the potential prognostic value of the indicated genes for the OS status (1:DECEASED or 0: LIVING) using timeROC package of R software (R.4.4.1). ROC curves comprise two parameters: true positive rate (sensitivity) and false positive rate (1-specificity) [39].

### Protein expression analysis

The subcellular localization and expression of the assessed markers were further verified at the protein levels by exploiting the Human Protein Atlas database (https://www.proteinatlas.org/) as previously described [40].

### Analysis of the expression levels of mitochondrial/OXPHOS genes in AML and healthy bone marrow-derived mononuclear cells

Exploiting Vizome database, the mRNA expression of the indicated mitochondrial/OXPHOS genes in biospecimens obtained from AML patients as well as that from healthy bone marrow-derived mononuclear cells (BMNCs) were downloaded from BeatAML.2 dataset [38].

### Analysis of the percent of blasts in the peripheral blood and bone marrow in AML patients with low and high expression levels of mitochondrial/OXPHOS genes

Based on the mRNA expression levels of mitochondrial/OXPHOS genes (mRNA expression z-scores relative to all samples – log RNA-Seq RPKM) in AML patients (OHSU dataset, Cancer Cell, 2022) [38], cBioPortal tool was exploited to inspect the percent of circulating blasts as well as blasts in the bone marrow biospecimens obtained from AML patients with low (z score ≤ -1) and high (z score ≥ 1) expression levels of the assessed genes.

### Analysis of the frequencies of genetic mutations in AML patients with low and high expression levels of mitochondrial/OXPHOS genes

Based on the mRNA expression levels of mitochondrial/OXPHOS genes (mRNA expression z-scores relative to all samples – log RNA-Seq RPKM), AML patients (OHSU dataset, Cancer Cell, 2022) were categorized into low (z score ≤ -1) and high (z score ≥ 1) expressing cohorts [38]. cBioPortal tool was then exploited to investigate the frequency of genetic mutations including missense mutations, inframe mutations, and truncating mutations in both cohorts of AML patients.

### Functional enrichment analysis of mitochondrial/OXPHOS co-expressing genes in primary human AML biospecimens

Genome-wide transcriptomics datasets of AML patients were obtained from cBioportal database (OHSU, Cancer Cell 2022) [38, 41]. Statistically significant co-expressed genes with a correlation value > 0.5 and adjusted *P* value ≤ 0.05 were selected for pathway enrichment. Indeed, we generated lists of the enriched genes associated with the indicated genes (NDUFA6, SDHA, CYB5A, SLC25A12, ETFB and CPT1A) to be exploited for functional annotation and enrichment analysis of gene ontology—biological processes 2023 using Enrichr [42].

### DepMap analysis of AML dependency on mitochondrial/OXPHOS genes

DepMap cancer dependency map identifies preferential dependencies of cancer cell lines [43, 44]. The dependency scores of human AML cell lines based on the indicated mitochondrial/OXPHOS genes were analysed using DepMap datasets of Sanger CRISPR Gene Effect (Project Score, Chronos) [43]. Lower Chronos score indicates a higher probability that the investigated gene is fundamental for that cancer cell line [43].

## Results

### Elevated levels of mitochondrial/OXPHOS genes correlate with poor prognosis of AML patients

**Fig. 1:**
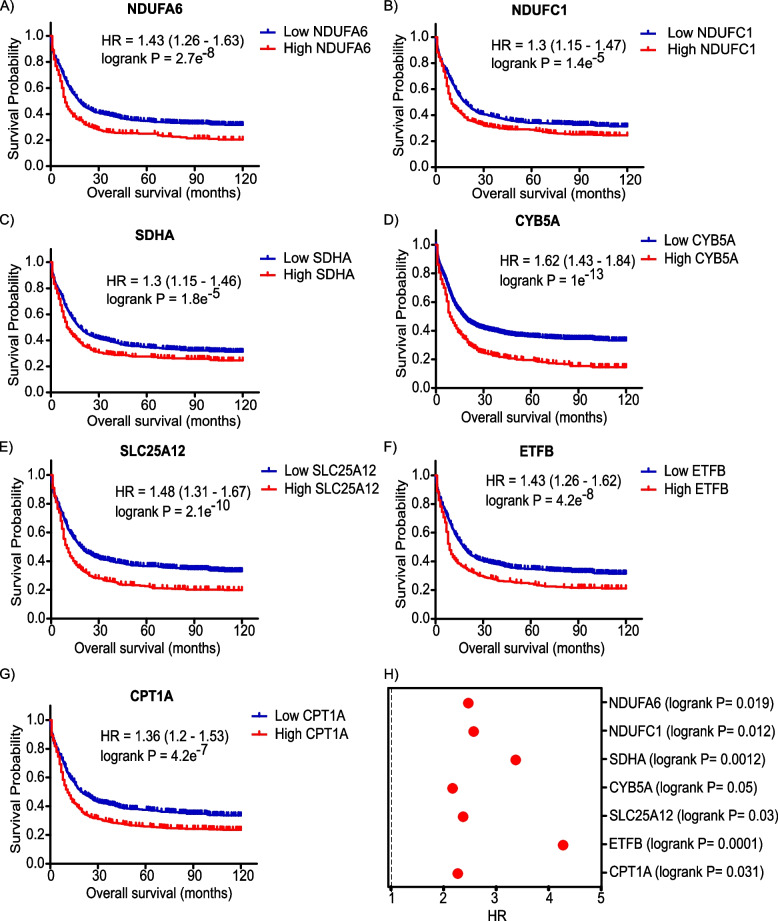
Elevated levels of mitochondrial/OXPHOS mediators correlate with poor prognosis of AML patients **A**-**G** Kaplan-Meier survival plots of **A** NADH:ubiquinone oxidoreductase subunit A6 (NDUFA6), **B** NADH:ubiquinone oxidoreductase subunit C1 (NDUFC1), **C** succinate dehydrogenase complex flavoprotein subunit A (SDHA), **D** cytochrome B5 type A (CYB5A), **E** solute carrier family 25 member 12 (SLC25A12), **F** electron transfer flavoprotein subunit beta (ETFB) and **G** carnitine palmitoyltransferase 1A (CPT1A)) in AML patients with a follow-up threshold of 120 months based on KM plotter AML dataset. Samples were divided into low (blue) and high (red) expression cohorts for each gene. Hazard ratio (HR) and log-rank *P* values are illustrated in each plot. **H** HR and log-rank *P* values of the prognostic potential of indicated genes in AML patients based on Gene Expression Profiling Interactive Analysis (GEPIA) database

Overall survival (OS) or the time from randomization to death is considered the gold standard endpoint in oncology clinical trials. To in silico test OXPHOS genes as candidate biomarkers for AML patients, we downloaded the list of human genes encoding proteins involved in OXPHOS (*n *= 200) from GSEA MSig database and exploited publically available transcriptional profiling datasets of AML patients (*n* = 734) with 10 years of follow-up using K-M plotter database [36]. Indeed, K-M analysis revealed that elevated transcript levels of NADH:ubiquinone oxidoreductase subunit A6 (NDUFA6), NADH:ubiquinone oxidoreductase subunit A8 (NDUFA8), NADH:ubiquinone oxidoreductase subunit A9 (NDUFA9), NADH:ubiquinone oxidoreductase subunit B5 (NDUFB5), NADH:ubiquinone oxidoreductase subunit B8 (NDUFB8), NADH:ubiquinone oxidoreductase subunit C1 (NDUFC1), NADH:ubiquinone oxidoreductase subunit S6 (NDUFS6), succinate dehydrogenase complex flavoprotein subunit A (SDHA), cytochrome B5 type A (CYB5A), solute carrier family 25 member 12 (SLC25A12), electron transfer flavoprotein subunit beta (ETFB), carnitine palmitoyltransferase 1A (CPT1A), phytanoyl-CoA 2-hydroxylase (PHYH), translocase of inner mitochondrial membrane 9 (TIMM9), cytochrome c oxidase subunit 7A2 (COX7A2), cytochrome c oxidase copper chaperone (COX11), cytochrome B5 reductase 3 (CYB5R3) and voltage dependent anion channel 2 (VDAC2) were significantly associated with poor OS in AML patients (log-rank *P* value < 0.05 and FDR = 1%) (Supplementary Table 1 and Fig. 1A-G). Notably, NDUFA6, SDHA, CYB5A, SLC25A12, ETFB and CPT1A remained significant when running a multivariate analysis including gender and treatment (untreated and chemotherapy treatment).

Event-free survival (EFS) is defined as the time from randomization to an event (as disease progression, treatment discontinuation or death). Yin and colleagues reported that EFS provided more precise assessment of the efficacy of drug therapy in AML patients [39]. Indeed, heightened levels of NDUFA6, NDUFC1, SDHA, CYB5A, SLC25A12, ETFB and CPT1A were associated with poor EFS in AML patients (Table 1), Next, we sought to validate the prognostic value of the above-mentioned mitochondrial/OXPHOS mediators for AML patients utilizing the Gene Expression Profiling Interactive Analysis (GEPIA) tool [37]. Genome-wide transcriptomics (RNA-Seq) datasets used by GEPIA is based on the UCSC Xena project [37]. GEPIA survival analysis confirmed that increased expression of NDUFA6, NDUFC1, SDHA, CYB5A, SLC25A12, ETFB and CPT1A significantly correlated with shorter OS of AML patients (Fig. 1H).

**Table 1:** Hazard ratios (HR) and log-rank *P* values of the assessed biomarkers in AML patients (*n *= 525) for event-free survival (EFS). Follow up threshold: 120 months

**Gene**	**Hazard ratio (HR) **	**Log-rank P**
NDUFA6	1.48 (1.18-1.85)	0.00058
NDUFC1	1.38 (1.1-1.73)	0.005
SDHA	1.45 (1.14-1.84)	0.0025
CYB5A	1.33 (1.05-1.69)	0.0194
SLC25A12	1.63 (1.29-2.07)	4.6e-^05^
ETFB	1.37 (1.07-1.75)	0.011
CPT1A	1.41 (1.13-1.77)	0.0023

*NDUFA6 NADH* ubiquinone oxidoreductase subunit A6, *NDUFC1 NADH* ubiquinone oxidoreductase subunit C1, *SDHA* succinate dehydrogenase complex flavoprotein subunit A, *CYB5A* cytochrome B5 type A, *SLC25A12* solute carrier family 25 member 12, *ETFB* electron transfer flavoprotein subunit beta, *CPT1A* carnitine palmitoyltransferase 1A, *GPX4* glutathione peroxidase 4

Furthermore, we took advantage of the BeatAML2 dataset which comprises real-world cohort of ~ 940 biospecimens with genomic, transcriptomic, and clinical annotations obtained from young (< 45 years) and older patients with de novo, transformed, or therapy-related AMLs [38]. Notably, upregulated levels of NDUFA6, SDHA, SLC25A12, ETFB and CPT1A were significantly associated with worse OS in AML patients (Table 2).

**Table 2:** Hazard ratios (HR) and log-rank *P* values of the assessed biomarkers in AML patients (BeatAML dataset, OHSU, Cancer Cell 2022) for overall survival (OS). Follow up threshold: 120 months

**Gene**	**Hazard ratio (HR)**	**Log-rank P**
NDUFA6	1.26 (1.01-1.58)	0.04
SDHA	1.4 (1.14-1.73)	0.0016
SLC25A12	1.27 (1.04-1.56)	0.0198
ETFB	1.39 (1.13-1.7)	0.0016
CPT1A	1.21 (1-1.48)	0.053
GPX4	1.3 (1.04-1.62)	0.02

*NDUFA6 NADH* ubiquinone oxidoreductase subunit A6,* SDHA *succinate dehydrogenase complex flavoprotein subunit A, *SLC25A12* solute carrier family 25 member 12, *ETFB *electron transfer flavoprotein subunit beta, *CPT1A* carnitine palmitoyltransferase 1A, *GPX4* glutathione peroxidase 4

Next, we exploited the Human Protein Atlas database to inspect the subcellular localization and expression of the assessed markers (Supplemental Table 2). Indeed, NDUFA6 and NDUFC1 encodes for accessory subunits of the mitochondrial complex I which promotes the transfer of electrons from NADH to the ETC [23, 45]. SDHA encodes for a major catalytic subunit of mitochondrial complex II [46]. In the citric acid cycle, complex II promotes the oxidation of succinate to fumarate [46]. CYB5A is a flavoprotein reductase which catalyzes electron transfer from NADH to target substrate [47]. SLC25A12 functions as a mitochondrial aspartate/glutamate carrier [48, 49]. ETFB is an electron-transfer-flavoprotein which transfers electrons between flavoprotein dehydrogenases and flavoprotein ubiquinone oxidoreductase [50]. CPT1A is the first rate-limiting enzyme of fatty acid oxidation which promotes the mitochondrial uptake of fatty acids [51]. In line with our analysis, SLC25A12, ETFB and CPT1A were reported to predict the prognosis of AML patients [49, 52].

In addition, time-dependent ROC and area under time dependent ROC curve (AUC) analysis revealed that the AUC values of NDUFA6 were 0.56, 0.54 and 0.72 at 12, 48 and 100 months respectively (Figure.S1A). The AUC values of SDHA were 0.58, 0.56 and 0.56 whereas those of SLC25A12 were 0.50, 0.49 and 0.55 at 12,24 and 48 months respectively (Figure.S1B-C). ETFB had AUC values of 0.56, 0.56 and 0.59 whereas CPT1A had AUC values of 0.51, 0.49 and 0.53 at 1,2 and 4 years respectively (Figure.S1D-E).

### Elevated levels of NDUFA6, SDHA, CYB5A, SLC25A12, ETFB and CPT1A are differentially associated with poor prognosis in AML patients with distinct mutations

Restricting K-M OS survival analyses to AML patients with distinct mutations revealed that upregulated levels of NDUFA6, SDHA and CPT1A were associated with unfavourable prognosis in mutated IDH1 AML patients (Table 3). Higher levels of NDUFA6 and CPT1A significantly correlated with poor OS of AML patients with IDH2 mutation. Elevated levels of NDUFA6, SDHA, CYB5A, SLC25A12, ETFB and CPT1A were associated with shorter OS of NPM1 and FLT3-ITD mutated AML patients (Table 3). Upsurged levels of SDHA, ETFB and CPT1A were linked to unfavourable prognosis of AML patients with FLT3-TKD mutation. Increased expression of SLC25A12 and CPT1A correlated with poor OS of AML patients with CEBPA mutation (Table 3).

**Table 3:** Hazard ratios (HR) and log-rank *P* values of the assessed biomarkers associated with poor prognosis in patients with mutated AMLs

Type of AML mutation (n)	Hazard ratio (HR)	Log-rank P
IDH1 mutation (*n* = 51)	NDUFA6: 3.28 (1.41–7.64)	0.0037
	SDHA: 2.1 (1.02- 4.32)	0.0405
	CPT1A: 2.68 (1.32–5.43)	0.0044
IDH2 mutation (*n* = 57)	NDUFA6: 2.45 (1.14–5.25)	0.0178
	CPT1A: 2.48 (1.25–4.92)	0.007
NPM1 mutation (*n* = 217)	NDUFA6:1.85 (1.18–2.88)	0.0059
	SDHA: 1.67 (1.16.2.41)	0.0057
	CYB5A: 1.49 (1.05–2.1)	0.0245
	SLC25A12: 1.79 (1.18–2.73)	0.0055
	ETFB: 1.54 (1.07–2.21)	0.0191
	CPT1A: 1.45 (1.02–2.06)	0.035
FLT3-ITD mutation (*n* = 191)	NDUFA6: 1.5 (1.07–2.1)	0.0188
	SDHA: 2.59 (1.85–3.64)	1e-^08^
	CYB5A: 1.5 (1.06–2.13)	0.0198
	SLC25A12: 1.58 (1.13–2.2) ETFB: 1.58 (1.12–2.23)	0.0065 0.0079
	CPT1A: 2.09 (1.5–2	9.9e-^06^
FLT3-TKD mutation (*n* = 79)	SDHA: 2.17 (1.14–4.14)	0.0163
	ETFB: 2.48 (1.31–4.7)	0.0041
	CPT1A: 3.41 (1.42–8.17)	0.003
CEBPA mutation (*n* = 52)	SLC25A12: 4.48 (1.32–15.18)	0.0084
	CPT1A: 2.57 (1.11–5.95)	0.023

*IDH1* isocitrate dehydrogenase 1, *IDH2* isocitrate dehydrogenase 2, *NPM1* nucleophosmin 1, *ITD* internal tandem duplication, *TKD* tyrosine kinase domain, *CEBPA *CCAAT/enhancer-binding protein alpha

### SLC25A12, ETFB and CPT1A are significantly overexpressed in AML biospecimens compared to healthy bone marrow-derived mononuclear cells

**Fig. 2:**
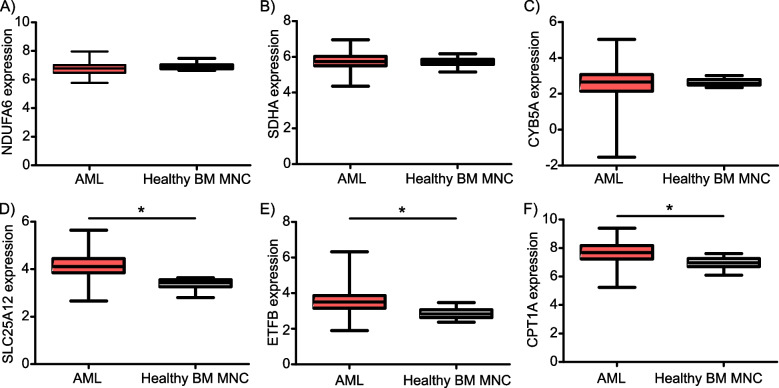
Solute carrier family 25 member 12 (SLC25A12), electron transfer flavoprotein subunit beta (ETFB) and carnitine palmitoyltransferase 1A (CPT1A) are significantly overexpressed in AML biospecimens compared to healthy bone marrow-derived mononuclear cells (BM MNC) **A**-**F** Transcript levels of the indicated genes (**A**: NADH:ubiquinone oxidoreductase subunit A6 (NDUFA6), **B**: succinate dehydrogenase complex flavoprotein subunit A (SDHA), **C**: cytochrome B5 type A (CYB5A), **D**: solute carrier family 25 member 12 (SLC25A12), **E**: electron transfer flavoprotein subunit beta (ETFB) and **F**: carnitine palmitoyltransferase 1A (CPT1A)) in AML biospecimens as well as healthy bone marrow-derived mononuclear cells obtained from BeatAML.2 dataset (38). *: *P* value ≤ 0.05 compared to healthy bone marrow-derived mononuclear cells

Next, we questioned whether the above-mentioned biomarkers are differentially expressed in primary human AML versus normal biospecimens. Exploiting BeatAML2 dataset revealed that there were no statistically significant differences in the mRNA levels of NDUFA6, SDHA, and CYB5A in biospecimens obtained from AML patients compared to healthy BMNCs (Fig. 2A-C). However, the transcript levels of SLC25A12, ETFB and CPT1A were significantly higher in AML compared to healthy BMNCs (Fig. 2D-F).

### AML patients with higher levels of CYB5A, SLC25A12 or CPT1A had significantly higher levels of circulating as well as engrafted blasts compared to low-expressing cohorts

**Fig. 3:**
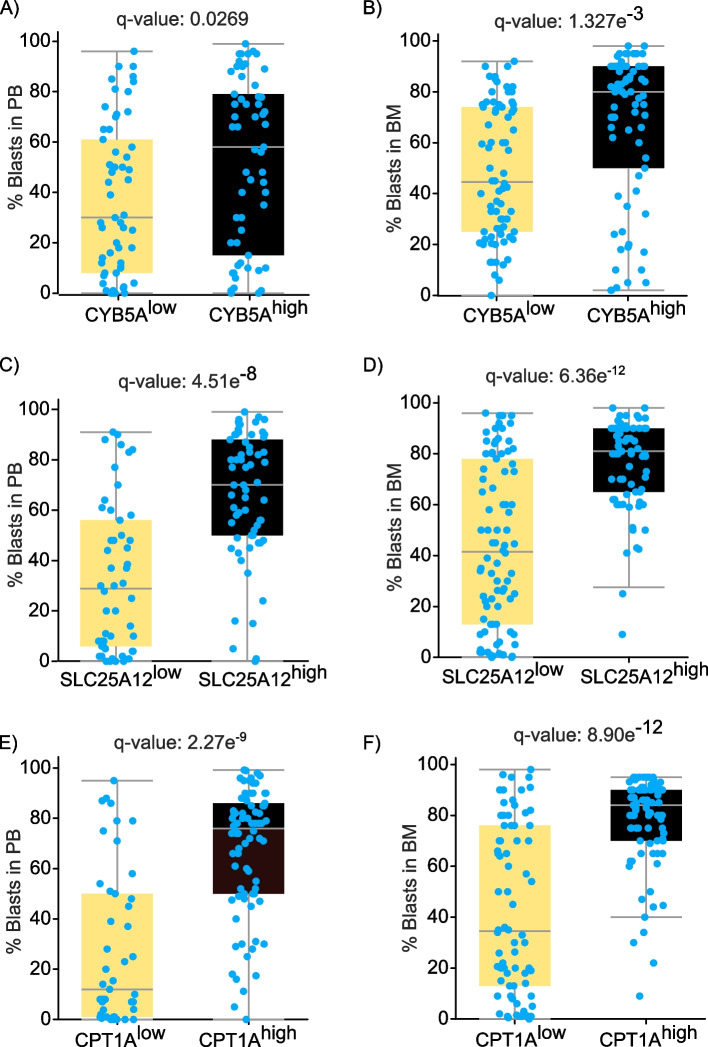
The percent of AML blasts in the peripheral blood as well as the bone marrow of AML patients with low and high expression levels of the indicated genes Based on the mRNA expression levels of the indicated genes (mRNA expression z-scores relative to all samples – log RNA-Seq RPKM) in AML patients (OHSU dataset, Cancer Cell, 2022) [38], cBioPortal tool was exploited to inspect the percent of circulating blasts as well as blasts in the bone marrow biospecimens of AML patients with low (z score ≤ -1) and high (z score ≥ 1) expression levels of the assessed genes. **A**, **B** The percent of circulating blasts (**A**) and blasts in the bone marrow (**B**) of cytochrome B5 type A (CYB5A)^low^ and CYB5A^high^ AML patients (BeatAML.2 dataset [38]). **C**, **D** The percent of circulating blasts (**C**) and blasts in the bone marrow (**D**) of solute carrier family 25 member 12 (SLC25A12)^low^ and SLC25A12^high^ AML patients. **E**, **F** The percent of circulating blasts (**E**) and blasts in the bone marrow (**F**) of carnitine palmitoyltransferase 1A (CPT1A)^low^ and CPT1A^high^ AML cohorts

Monitoring the percent of blasts in the peripheral blood as well as bone marrow is critical for the diagnosis, prognosis, and therapy of AML patients [2, 53, 54]. Accordingly, we inspected whether AML patients with low and high levels of biomarkers as NDUFA6, SDHA, CYB5A, SLC25A12, ETFB and CPT1A have differential leukemic burden in their peripheral blood and bone marrow biospecimens. To this end, we categorized AML patients (BeatAML2, OHSU, Cancer 2022 dataset) according to the mRNA expression of the assessed genes (z-scores relative to all samples – log RNA-Seq RPKM) into low (z score ≤ -1) and high (z score ≥ 1) expressing cohorts. Statistical analyses of the clinical attributes of low- and high-expressing cohorts are summarized in Supplemental Table 3. Notably, CYB5A^low^ AML cohort had significantly lower percent of blasts in both the peripheral blood as well as bone marrow compared to CYB5A^high^ AML cohort (Fig. 3A-B). Similar findings were also noted in SLC25A12^low^ versus SLC25A12^high^ and in CPT1A^low^ versus CPT1A^high^ AML cohorts (Fig. 3C-F). It is worth mentioning that the current regimen and biospecimen type of SLC25A12^low^ cohort (69% bone marrow aspirate and 31% peripheral blood) were statistically significant from that of SLC25A12^high^ cohort (45.13% bone marrow aspirate, 7.01% leukapheresis and 47.79% peripheral blood) (Supplemental Table 3). Otherwise, the age at biospecimen collection, ethnicity category, sex, specimen type and current regimen were not statistically different among other low- and high-expressing cohorts (Supplemental Table 3).

### Prevalence of genetic mutations in AML patients with low and high levels of mitochondrial/OXPHOS genes

**Fig. 4:**
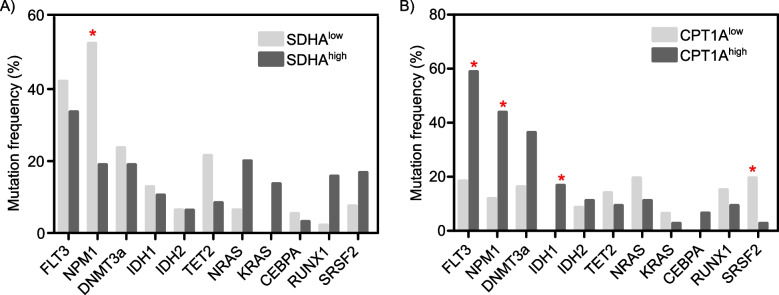
Frequency of genetic mutations in AML patients with low and high levels of the indicated genes **A, B** Mutation frequencies of the indicated genes in AML patients with low and high expression levels of succinate dehydrogenase complex flavoprotein subunit A (SDHA) (**A**) and carnitine palmitoyltransferase 1A (CPT1A) (**B**). *: Adjust *P* value ≤ 0.05

Given the prevalence of genetic mutations in AML, we examined the correlation between the transcriptional levels of the forementioned genes (low versus high expressing cohorts) and AML mutations using the cBioPortal tool [41]. Intriguingly, NPM1 and serine/arginine-rich splicing factor 2 (SRSF2) mutations were higher in SDHA^low^ and CPT1A^low^ AML cohorts respectively (Fig. 4A-B). In contrast, FLT3-ITD, NPM1 and IDH1 mutations were more common in CPT1A^high^ AML patients (Fig. 4B).

### Functional prediction and pathway enrichment analysis of mitochondrial/OXPHOS genes in primary human AML biospecimens

**Fig. 5:**
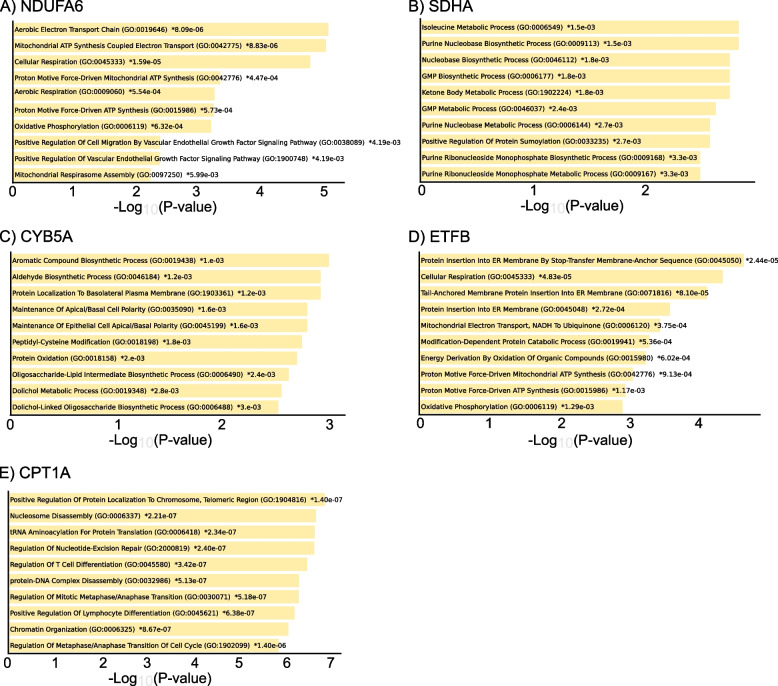
Gene ontology of the biological process of mitochondrial/OXPHOS co-expressed genes in AML patients **A-E**) Bar chart of top enriched terms from the GO_Biological_Process_2023 gene set library which are significantly co-expressed with NADH: ubiquinone oxidoreductase subunit A6 (NDUFA6) (**A**), succinate dehydrogenase complex flavoprotein subunit A (SDHA) (**B**), cytochrome B5 type A (CYB5A) (**C**), electron transfer flavoprotein subunit beta (ETFB) (**D**) and carnitine palmitoyltransferase 1A (CPT1A) (**E**) in biospecimens obtained from AML patients (OHSU Cancer Cell 2022 dataset). The cut-off for Spearman’s correlation is > 0.5 and adjusted *P* value ≤ 0.05. The top 10 enriched terms for the input gene set are displayed based on the -log_10_ (*P* value), with the actual *P* value illustrated next to each term. The term at the top has the most significant overlap with the input query gene set

We examined the genes which are co-expressed with mitochondrial/OXPHOS genes using the cBioPortal tool [38, 41]. The expression of NDUFA6 positively correlated with the upregulation of several genes including succinate dehydrogenase complex iron sulfur subunit B (SDHB), cysteine rich with EGF like domains 2 (CRELD2), adaptor related protein complex 2 subunit sigma 1 (AP2S1), HIG1 hypoxia inducible domain family member 1A (HIGD1A), cytochrome C oxidase subunit 7B (COX7B), proteasome maturation protein (POMP), myosin 1C (MYO1C), mitochondrial ribosomal protein S15 (MRPS15), NADH: ubiquinone oxidoreductase subunit B9 (NDUFB9), calmodulin binding transcription activator 1 (CAMTA1), G protein regulated inducer of neurite outgrowth 1 (GPRIN1) and BolA family member 3 (BOLA3). Gene Ontology (GO) enrichment analysis of NDUFA6 co-expressing genes showed the enrichment of distinct biological processes (BP) as mitochondrial ATP synthesis coupled electron transport, cellular respiration, oxidative phosphorylation, positive regulation of cell migration by vascular endothelial growth factor signalling pathway, positive regulation of vascular endothelial growth factor signalling pathway and mitochondrial respirasome assembly (Fig. 5A).

On the other hand, the expression of SDHA positively correlated with the expression of acetyl-CoA acetyltransferase 1 (ACAT1), SUMO1 activating enzyme subunit 1 (SAE1), ATP synthase membrane subunit C locus 3 (ATP5MC3), TNF receptor associated protein 1 (TRAP1), guanine monophosphate synthase (GMPS) and protein phosphatase 1 catalytic subunit gamma (PPP1CC). GO BP analysis of SDHA co-expressing genes highlighted the enrichment of isoleucine metabolic process, purine nucleibase biosynthetic process, ketone body metabolic process and positive regulation of protein sumoylation (Fig. 5B).

GO BP enrichment analysis of CYB5A co-expressed genes revealed their association with aromatic compound biosynthetic process, aldehyde biosynthetic process, protein localization to basolateral plasma membrane, maintenance of apical/basal cell polarity, peptidyl-cysteine modification, protein oxidation, oligosaccharide-lipid intermediate biosynthetic process, and dolichol metabolic process (Fig. 5C).

ETFB co-expressed genes were rather enriched with biological processes as protein insertion into ER membrane by stop-transfer membrane-anchor sequence, cellular respiration, mitochondrial electron transport, NADH to ubiquinone and energy derivation by oxidation of organic compounds (Fig. 5D).

GO enrichment analysis of CPT1A co-expressed genes revealed the enrichment of BP as positive regulation of protein localization to chromosome, telomeric region, nucleosome disassembly, tRNA aminoacylation for protein translation, regulation of nucleotide-excision repair, regulation of mitotic metaphase/anaphase transition and chromatin organization (Fig. 5E).

### Differential dependency of AML cells on mitochondrial/OXPHOS genes

Based on the above-mentioned findings, we questioned whether human AML cells are dependent on the evaluated mitochondrial/OXPHOS genes. At genome-wide scale, the CRISPR–Cas9 system objectively identifies genes which are indispensable for the proliferation and survival of cancer cells including AML [44, 55]. To this end, we exploited the Cancer Dependency Map database (which comprises large-scale functional genomics profiling using CRISPR loss-of-function screens) to investigate the potential dependency of AML cells on mitochondrial/OXPHOS genes. Dempster and colleagues developed Chronos model which addressed several limitations associated with other models including sgRNA efficacy, variable screen quality and copy number bias [43]. Negative Chronos scores indicate slower growth of cancer cells [43].

**Fig. 6:**
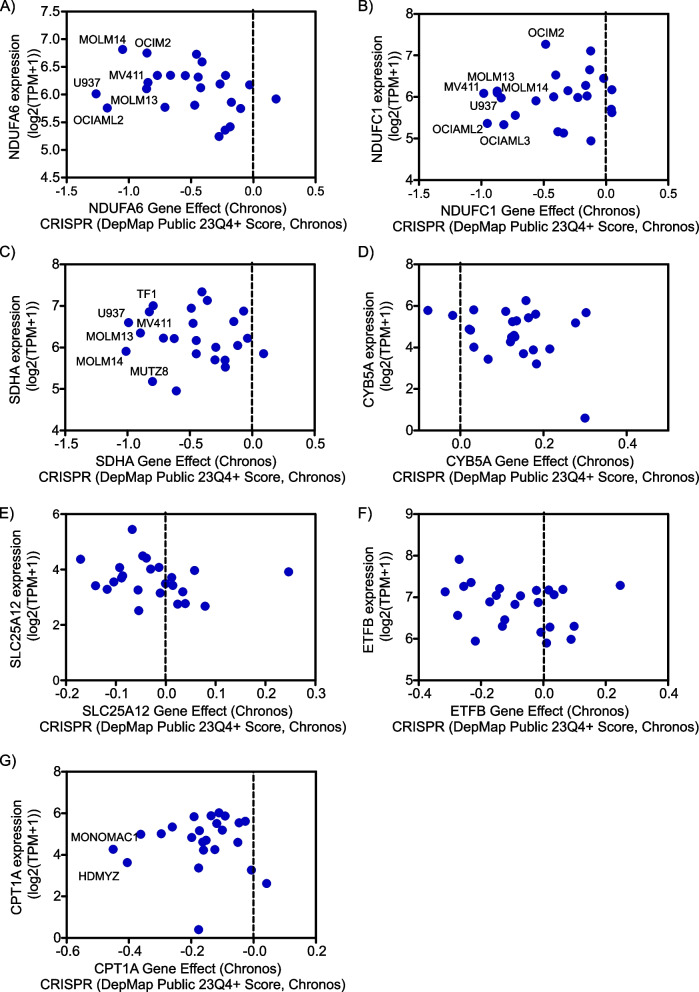
Differential dependencies of AML cells on mitochondrial/OXPHOS genes **A**-**G** Dot plot depicting the gene dependency effect (CRISPR, DepMap Public 23Q4+ Score, Chronos) of AML cells on the indicated mitochondrial/OXPHOS genes (**A**: NADH : ubiquinone oxidoreductase subunit A6 (NDUFA6), **B**: NADH : ubiquinone oxidoreductase subunit C1 (NDUFC1), **C**: succinate dehydrogenase complex flavoprotein subunit A (SDHA), **D**: cytochrome B5 type A (CYB5A), **E**: solute carrier family 25 member 12 (SLC25A12), **F**: electron transfer flavoprotein subunit beta (ETFB) and **G**: carnitine palmitoyltransferase 1A (CPT1A)) plotted on the X-axis versus their corresponding mRNA expression level (log2(TPM+1) plotted on the Y-axis

Indeed, AML cells exhibited heterogeneous responses to CRISPR-mediated genetic pertubation of OXPHOS genes which encode for subunits of the OXPHOS complexes: I (NDUFA6 and NDUFC1) and II (SDHA) (Fig. 6A-C). Of note, AML cell lines with FLT3-ITD mutations (as MV4-11, MOLM13 and MOLM14) [5] were more vulnerable to the genetic depletion of NDUFA6, NDUFC1 and SDHA (Fig. 6A-C). In contrast, modest dependencies of AML cells were observed for CRISPR KO of CYB5A, SLC25A12, ETFB and CPT1A (Fig. 6D-G). Overall, these findings highlight the therapeutic potential of targeting mitochondrial/OXPHOS dependent/addicted AML cells.

### Heightened expression of glutathione peroxidase 4 (GPX4) is associated with higher percent of circulating and engrafted blasts of AML patients and DNMT3a mutant AML cells are dependent on GPX4

**Fig. 7:**
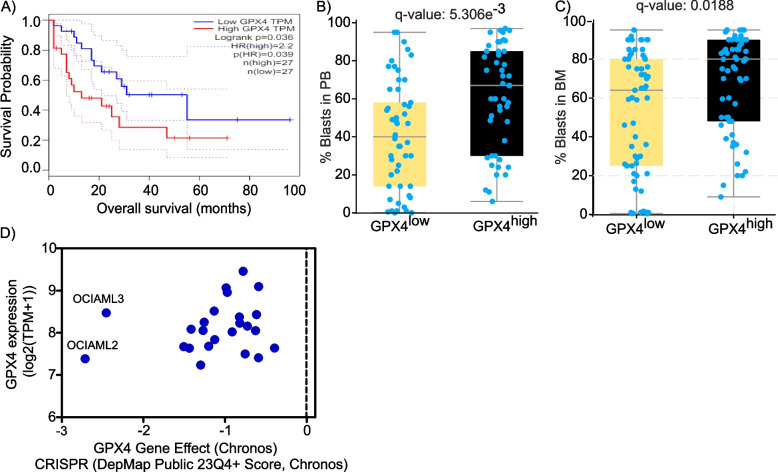
Increased expression of glutathione peroxidase 4 (GPX4) is associated with higher percent of circulating and engrafted blasts as well as poor prognosis of AML patients and DNMT3a mutant AML cells are dependent on GPX4 **A** Kaplan–Meier survival curve depicting the hazard ratio (HR) and log**-**rank *P* values of the prognostic potential of GPX4 gene in AML patients based on Gene Expression Profiling Interactive Analysis (GEPIA) database. **B**, **C** The percent of circulating blasts (**B**) and blasts in the bone marrow (**C**) of GPX4^low^ and GPX4^high^ AML patients (BeatAML.2 dataset (38)). **D** Dot plot depicting the gene dependency effect (CRISPR, DepMap Public 23Q4 + Score, Chronos) of AML cells on GPX4 plotted on the X-axis versus their corresponding mRNA expression level (log2(TPM + 1) plotted on the Y-axis

Glutathione peroxidase 4 (GPX4) is included in the geneset of HALLMARK_OXIDATIVE_PHOSPHORYLATION downloaded from GSEA MSigDB. As an antioxidant, GPX4 promotes the reduction of hydrogen peroxide, organic hydroperoxides and lipid hydroperoxides [56]. Km plotter database and GEPIA survival analyses showed controversial results in terms of the prognostic value of GPX4 expression in AML patients. Unlike Km plotter, the survival analyses of GEPIA and BeatAML.2 datasets demonstrated that elevated GPX4 levels are significantly associated with worse OS of AML patients (Fig. 7A and Supplemental Table 2). The AUC values of the ROC curve of GPX4 were 0.51, 0.52 and 0.62 at 2, 4 and 6 years respectively (Figure.S1F). GPX4^high^ AML cohort had significantly higher percent of blasts in both the peripheral blood as well as bone marrow compared to GPX4^low^ AML cohort (Fig. 7B-C).

Several patient-derived AML cell lines were evidently susceptible to CRISPR KO of GPX4 (Fig. 7D). Notably, OCIAML2 and OCIAML3 AML cell lines were preferentially responsive to the genetic ablation of GPX4. It is worth noting that OCI-AML2 and OCI-AML3 are the only known human AML cell lines with the DNMT3A mutation. Altogether, these findings underscore the therapeutic potential of targeting GPX4 for AML therapy.

## Discussion

The present study underscored the prognostic value of mitochondrial/OXPHOS genes as NDUFA6, SDHA, SLC25A12, ETFB CPT1A and GPX4 in AML patients. Consistent with the present findings, breast cancer patients with high NDUFA6 levels had shorter OS [57]. Elevated levels of NDUFC1 also predicted unfavourable prognosis of hepatocellular carcinoma patients [32]. In line with this study, elevated levels of SLC25A12, ETFB and CPT1A were associated with poor prognosis of AML patients [49, 52]. High CPT1A levels were associated with worse OS of nasopharyngeal carcinoma patients post-radiotherapy [51]. Inspecting BeatAML.2 dataset, the present study also showed that CYB5A^high^, SLC25A12^high^ and CPT1A^high^ AML patients had significantly higher levels of circulating as well as engrafted blasts compared to CYB5A^low^, SLC25A12^low^ and CPT1A^low^ AML patients respectively. CYB5A reduces ferric hemoglobin to ferrous hemoglobin, which is required for stearoyl-CoA-desaturase activity [47]. Stearoyl coA desaturase converts saturated fatty acids into monounsaturated fatty acids. Intriguingly, high expression of stearoyl-coA-destaurase is associated with poor prognosis of gastric cancer patients [58].

More specifically, this study found that NPM1 W288Cfs*12 frameshift insertion mutations are prevalent in SDHA^low^ and CPT1A^high^ AML patients. NPM1 mutant insertions as W288Cfs*12 (tryptophan 288 to cysteine frameshift at exon 12) result in a change in the C-terminal domain (CTD) amino acid sequence which is required for nucleolar localization signalling [59]. The present study also shed light on the prevalence of SRSF2 P95H missense mutation in CPT1A^low^ AML patients which is located in the RNA binding domain of the SRSF2 splicing factor protein and leads to distorted RNA binding of SRFS2 (56). Likewise, the frequently occurring IDH1 R132H mutation in CPT1A^low^ AML patients is located in the catalytic site of the IDH1 protein which converts α-ketoglutarate to 2-hydroxyglutarate oncometabolite [1, 8, 60].

The present study also shed light on the heterogeneous responsiveness of AML cells to genetic depletion of OXPHOS genes as NDUFA6, NDUFC1 and SDHA. Knocking down NDUFC1 evidently impeded the proliferation, cell cycle progression, migration and invasion and triggered apoptotic cell death of hepatocellular carcinoma cells [32] Notably, FLT3-ITD^+^ AML cell lines (MV4-11, MOLM13 and MOLM14) [5] were more suseptible to genetic intereference with subunits of mitochondiral complex I (NDUFA6 and NDUFC1) and mitochondrial complex II (SDHA). Consistently, Baccelli and colleagues reported that AML addiction on mitochondrial complex I activity was strongly associated with the presence of NPM1 and FLT3-ITD mutations as well as those mutations affecting DNA methylation genes (as DNMT3a, IDH1, IDH2, and TET2) [25]. Intriguingly, IACS-010759 (an OXPHOS complex I inhibitor) synergized with quizartinib (a FLT3 inhibitor) on FLT3-ITD^+^ and FLT3-ITD^−^ AMLs [61]. Despite its promising preclinical activity, Phase I study reported the narrow therapeutc index and dose-limiting toxicities of IACS-010759 when evaluated in patients with solid tumors as well as AML [62]. Erdem and colleagues demonstrated that FLT3-ITD^+^ AML patients had overactivated mitochondrial ETC complex II [57]. Although abrogating mitochondrial ETC complex II triggered apoptotic cell death, FLT3-ITD^+^ AMLs adapted by importing lactate to be exploited for mitochondrial respiration. Blocking lactate transport evidently augmented the anti-AML activity of ETC complex II inhibitors [57]. Blunting CPT1A sensitized nasopharyngeal cancer cells to radiotherapy via triggering mitochondrial apoptosis [51].

Compared with GPX4^low^ AML patients, GPX4^high^ cohort had higher percent of blasts both in the peripheral blood as well as bone marrow. Wei and colleagues showed that AML patients with high expression of GPX1, GPX3, GPX4, and GPX7 were associated with worse prognosis [58]. In accordance with the present findings, pharmacological as well as genetic suppression of GPX4 induced ferroptosis (a non-apoptotic cell death triggered by iron-dependent lipid peroxidation) in AML cells [58]. In this study, DNMT3a mutant AML cell lines (OCIAML2 and OCIAML3) were dependent on GPX4. Indeed, further mechanistic studies are needed to decipher the molecular basis underlying the preferential sensitivity of these AML cell lines and examine whether this is linked to DNMT3a mutation.

Altogether, the present study is a comprehensive and systematic bioinformatic analysis based on multiple databases and datasets of AML patients. However, further in vitro and in vivo validation of these findings is warranted.

## Conclusions

To the best of our knowledge, this is the first study which systematically investigated the potential value of OXPHOS genes which could serve as prognostic biomarkers for AML patients. Moreover, this study highlighted differential dependencies of AML cells on NDUFA6, SDHA, CYB5A, SLC25A12, ETFB, and CPT1A which could rationally guide personalized therapy of AML patients. Nonetheless, caution is warranted to address the feasibility and safety profile of mitochondrial ETC complex I inhibitors before further advancing their preclinical and clinical development for AML therapy.

## Supplementary Information

Supplementary Material 1.

Supplementary Material 2: Figure S1. Area under the curve (AUC) of receiver operating characteristic (ROC) curves of the indicated genes in biospecimens obtained from AML patients (BeatAML.2 cBioportal dataset (OHSU, Cancer Cell, 2022). A-F) ROC curves and AUC of NADH:ubiquinone oxidoreductase subunit A6 (NDUFA6) (A), succinate dehydrogenase complex flavoprotein subunit A (SDHA) (B), solute carrier family 25 member 12 (SLC25A12) (C), electron transfer flavoprotein subunit beta (ETFB) (D), carnitine palmitoyltransferase 1A (CPT1A) (E) and glutathione peroxidase 4 (GPX4) (F) at the indicated time points of overall survival.

## Data Availability

No datasets were generated or analysed during the current study.

## Abbreviations

COX7A2: Cytochrome c oxidase subunit 7A2
COX11: Cytochrome c oxidase copper chaperone
CPT1A: Carnitine palmitoyltransferase 1A
CYB5A: Cytochrome B5 type A
CYB5R3: Cytochrome B5 reductase 3
ETC: Electron transport chain
ETFB: Electron transfer flavoprotein subunit beta
GPX4: Glutathione peroxidase 4
GEPIA: Gene expression profiling interactive analysis
NDUFA6: NADH:ubiquinone oxidoreductase subunit A6
NDUFA8: NADH:ubiquinone oxidoreductase subunit A8
NDUFA9: NADH:ubiquinone oxidoreductase subunit A9
NDUFB5: NADH:ubiquinone oxidoreductase subunit B5
NDUFB8: NADH:ubiquinone oxidoreductase subunit B8
NDUFC1: NADH:ubiquinone oxidoreductase subunit C1
NDUFS6: NADH:ubiquinone oxidoreductase subunit S6
OS: Overall survival
PHYH: Phytanoyl-CoA 2-hydroxylase
SDHA: Succinate dehydrogenase complex flavoprotein subunit A
SLC25A12: Solute carrier family 25 member 12
TIMM9: Translocase of inner mitochondrial membrane 9
VDAC2: Voltage dependent anion channel 2
