# Supplementary material for: OXPHOS mediators in acute myeloid leukemia patients: Prognostic biomarkers and therapeutic targets for personalized medicine
Source: World J Surg Oncol. 2024 Nov 12;22:298. doi: 10.1186/s12957-024-03581-5 (PMC11559054; doi:10.1186/s12957-024-03581-5)
Supplement: Supplementary file 2 — Supplementary Material 2: Figure S1. Area under the curve (AUC) of receiver operating characteristic (ROC) curves of the indicated genes in biospecimens obtained from AML patients (BeatAML.2 cBioportal dataset (OHSU, Cancer Cell, 2022). A-F) ROC curves and AUC of NADH:ubiquinone oxidoreductase subunit A6 (NDUFA6) (A), succinate dehydrogenase complex flavoprotein subunit A (SDHA) (B), solute carrier family 25 member 12 (SLC25A12) (C), electron transfer flavoprotein subunit beta (ETFB) (D), carnitine palmitoyltransferase 1A (CPT1A) (E) and glutathione peroxidase 4 (GPX4) (F) at the indicated time points of overall survival. [file 12957_2024_3581_MOESM2_ESM.zip › Legend of Supplementary Figure.docx]

**Legends of Supplementary Figure**

**Figure.S1. Area under the curve (AUC) of receiver operating characteristic (ROC) curves of the indicated genes in biospecimens obtained from AML patients (BeatAML.2 cBioportal dataset (OHSU, Cancer Cell, 2022).** A-F) ROC curves and AUC of NADH:ubiquinone oxidoreductase subunit A6 (NDUFA6) (A), succinate dehydrogenase complex flavoprotein subunit A (SDHA) (B), solute carrier family 25 member 12 (SLC25A12) (C), electron transfer flavoprotein subunit beta (ETFB) (D), carnitine palmitoyltransferase 1A (CPT1A) (E) and glutathione peroxidase 4 (GPX4) (F) at the indicated time points of overall survival.
